# Toxic metal levels in children residing in a smelting craft village in Vietnam: a pilot biomonitoring study

**DOI:** 10.1186/1471-2458-14-114

**Published:** 2014-02-04

**Authors:** Alison P Sanders, Sloane K Miller, Viet Nguyen, Jonathan B Kotch, Rebecca C Fry

**Affiliations:** 1 Department of Environmental Sciences and Engineering, Gillings School of Global Public Health, University of North Carolina - Chapel Hill, Chapel Hill, North Carolina, USA; 2 Department of Maternal and Child Health, Gillings School of Global Public Health, University of North Carolina - Chapel Hill, Chapel Hill, North Carolina, USA; 3 Curriculum in Toxicology, School of Medicine, University of North Carolina - Chapel Hill, Chapel Hill, North Carolina, USA; 4 Lineberger Comprehensive Cancer Center, School of Medicine, University of North Carolina - Chapel Hill, Chapel Hill, North Carolina, USA

## Abstract

**Background:**

In Vietnam, environmental pollution caused by small-scale domestic smelting of automobile batteries into lead ingot is a growing concern. The village of Nghia Lo is a smelting craft village located roughly 25 km southeast of Hanoi in the Red River Delta. Despite the concern of toxic metal exposure in the village, biomonitoring among susceptible populations, such as children, has not been previously conducted. The aim of this study was to determine the body burden of toxic metals in children residing in a smelting craft village.

**Methods:**

Twenty children from Nghia Lo, Vietnam, ages 18 months to four years were selected for capillary whole blood and toenail biomonitoring. Whole blood lead levels (BLLs) were measured using a portable lead analyzer, and toenail levels of arsenic, cadmium, chromium, lead, manganese, and mercury were analyzed with inductively coupled plasma-mass spectrometry.

**Results:**

The findings show that all of the 20 children had detectable BLLs, and every child had levels that exceeded the Centers for Disease Control and Prevention guideline level of 5 μg/dL. Eighty percent of tested subjects had BLLs higher than 10 μg/dL. Five children (25%) had BLLs greater than 45 μg/dL, the level of recommended medical intervention. In addition to blood lead, all of the children had detectable levels of arsenic, cadmium, chromium, lead, manganese, and mercury in toenail samples. Notably, average toenail lead, manganese, and mercury levels were 157 μg/g, 7.41 μg/g, and 2.63 μg/g respectively, well above levels previously reported in children. Significant Spearman’s rank correlations showed that there were relationships between blood and toenail lead levels (r = 0.65, p < 0.05), toenail levels of lead and cadmium (r = 0.66, p < 0.05), and toenail levels of manganese and chromium (r = 0.72, p < 0.001). Linear regression showed that reducing the distance to the nearest active smelter by half was associated with a 116% increase in BLL (p < 0.05).

**Conclusions:**

The results suggest that children in battery recycling and smelting craft villages in Vietnam are co-exposed to toxic metals. There is an urgent need for mitigation to control metal exposure related to domestic smelting.

## Background

Widespread environmental lead (Pb) exposure contributes to adverse neurodevelopmental outcomes in children, which is associated with substantial economic losses [1,2]. The associated economic losses are estimated up to $319 billion per year in the United States [1]. The detrimental neurodevelopmental effects of both prenatal and childhood lead exposure include decreased intelligence quotient (IQ) and cognitive function, diminished attention span, academic proficiency, fine-motor control, and visual-motor control [3,4]. Children are especially at risk for Pb exposure due to increased hand to mouth activity, increased exposure to soils and household dusts containing Pb, and higher relative gastrointestinal absorption of Pb [5-7]. These factors can result in elevated blood lead levels (BLLs) in children [5,6] and a greater adverse effect on the developing nervous system [3]. Specifically, a 10 μg/dL average increase in children’s blood Pb concentration is associated with a decline in 1.9 to 3.2 IQ points [1].

Pb exposure often coincides with other toxic metal exposures due to co-contamination of air, soil, or water supplies by industrial activities [8-12]. However, there have been limited studies of the interactive effects of toxic metals resulting from co-exposure in humans. Co-exposure to toxic metals is associated with adverse health outcomes including birth defects and various types of cancer [10,11]. In addition, toxic metal exposure in childhood has been reported to decrease neuropsychological function, IQ, and overall academic achievement [13-15]. Lending support, mixed exposure to metals such as arsenic (As), cadmium (Cd), mercury (Hg), and Pb has been shown to have greater toxicological effects in animal studies than exposure to a single metal [16-20].

The Red River Delta of Vietnam is approximately 21,000 km^2^ and has a population of nearly 20 million [21]. Co-exposure to toxic metals has been previously documented in this region due to a variety of sources, including occupational exposures due to local craft villages [22,23] as well as naturally-occurring metals in drinking water [24-27]. A craft village is a community in which multiple households participate in the production of a particular good such as textiles, construction materials, recycled metal, paper, or plastic. The recycling of Pb-acid batteries is a lucrative process for local communities; however, it can result in massive Pb toxicity [28,29]. BLLs in children living in battery recycling communities have been measured as high as 613 μg/dL [28]. The recommended blood advisory level in the United States is 5 μg/dL for children [30]. Of concern, there is a lack of information on the environmental and broader health impacts of craft villages, particularly with respect to mixed toxic metal exposure.

In this pilot study we set out to determine the body burden of blood Pb in children residing in a smelting craft village in Nghia Lo, Vietnam. We also characterized levels of other toxic metals including As, Cd, chromium (Cr), manganese (Mn), Hg, and Pb in toenail samples. This pilot study begins to characterize children’s toxic metal exposure in smelting craft villages in Vietnam.

## Methods

### Study population

The Nghia Lo village, Vietnam was selected for study due to local Pb smelting practices and prior evidence of environmental metal contamination. Nghia Lo is a rural community located roughly 25 km southeast of Hanoi in the Commune of Chi Dao, Hung Yen Province. The village population includes 1,800 individuals and 533 total households. In 2011, there were 211 children under five years of age. This cross-sectional pilot study selected 20 children who lived in closest proximity to domestic Pb smelters and/or had parents whose work included grinding the plastic cases of automobile batteries. This study was reviewed and approved by the Institutional Review Boards at the University of North Carolina (#11-1371) and the Institute of Child Health Research in Vietnam. Parents and legal guardians consented for all children participating in this study. Capillary whole blood and toenail samples for metal biomonitoring analysis were collected from all participants. Demographic information including child’s age and sex were provided by the parents during an interview following the signing of the informed consent and preceding biological specimen collection. Families with children whose BLLs exceeded 45 μg/dL were offered chelation treatment at the project’s expense.

### Distance to exposure

A portable global positioning system (GPS) device was used to collect the geographic coordinates of participants’ residences, smelters, plastic refineries, and local schools. GPS coordinates were visualized in ESRI ArcGIS™ software Version 10.0 (Redlands, CA). Shapefiles of Vietnam were obtained from OpenStreetMap© contributors (openstreetmap.org). Distance to the nearest active smelter was calculated for each child.

### Capillary whole blood collection and analysis

Children’s fingers were prepared with an alcohol wipe according to the Centers for Disease Control and Prevention (CDC) guidelines and samples were collected into capillary tubes following a finger prick. Capillary whole blood was collected into metals-free phlebotomy tubes by a registered medical doctor. Samples were immediately analyzed for Pb content using our portable LeadCare® II Analyzer (Magellan Diagnostics, Billerica, MA). The LeadCare® II is the only CLIA-waived device available to test for Pb exposure at the point of care. The LeadCare® II is currently used in clinics across the United States including Departments of Public Health; Women, Infants and Children (WIC)s clinics; and Head Start Programs. The portable system provides rapid quantification of fresh whole blood Pb levels in a rural setting. Control spikes and capillary whole blood samples were processed according to the manufacturer’s protocol. Detectable BLLs measured by the system ranged between 3.3 and 69 μg/dL. Pb was the only metal measured in the children’s capillary whole blood.

### Toenail collection and analysis

Toenails were collected by a registered nurse using a clean nail clipper. Toenail clippings were transported to the United States and metals analyses were performed at RTI International, Research Triangle Park, North Carolina. Prior to chemical analysis, nails were stringently washed with double distilled water and acetone to remove debris and any external trace metals. The toenails were allowed to dry at room temperature overnight in a sterile hood. The cleaned toenail samples were stored at room temperature in 1.5 mL tubes until weighing and chemical analysis.

Toenail samples from 20 children were analyzed for six metals using inductively coupled plasma–mass spectrometry (ICP-MS). The metals included As, Cd, Cr, Pb, Mn, Hg. The toenails were extracted using a modified version of Method 3050B (USEPA, 1996). Each sample was spiked with 10 μL of 1000 ppm gold stock solution in order to retain mercury for measurement. Next, 0.5 mL nitric acid (HNO_3_), 0.1 mL hydrochloric acid (HCl), and 1 mL deionized water were added to the samples. The samples were heated at 95°C loosely capped to allow reflux for one hour. The samples were cooled, 1 mL of 30% hydrogen peroxide was added, and the samples were heated at 95°C for 15 min. The samples were cooled, 0.4 mL of HCl was added, and the samples were heated at 95°C for an additional 15 min. The samples were brought to a final volume of 10 mL using deionized water and vortex mixed. Samples were analyzed using a Thermo X-Series II ICP-MS equipped with a dual gas collision cell. The limit of detection (LOD) among toenail metals varied for each individual because the available mass differed by sample, and a higher sample mass resulted in a lower LOD.

### Statistical analysis

Statistical analysis was conducted using SAS 9.3 (SAS Institute Inc., Cary, North Carolina). Descriptive statistics were calculated for 20 children who provided capillary whole blood and toenail samples. Three samples for which BLLs exceeded the maximum quantifiable level of the LeadCare® II Analyzer of 69 μg/dL were excluded from further analysis with toenail metal levels or other covariates, including sex, age, and distance to the nearest active smelter. No toenail metal levels were below their respective LOD. Spearman’s correlation coefficient estimates among metal pairs and corresponding p-values were calculated. Toenail and blood metal levels were natural-log transformed to address the non-normal distribution of metal concentrations. Nearest distance to an active smelter (km) was also natural-log transformed. Unadjusted linear regression was performed to test the association between each blood or toenail metal level and child’s age (treated as a continuous variable), sex (classified as a dichotomous variable with female serving as the referent group), or distance to nearest active smelter (treated as a continuous variable). Beta estimates and 95% confidence intervals are presented, with statistical significance defined at alpha less than 0.05.

## Results and Discussion

**Figure 1:**
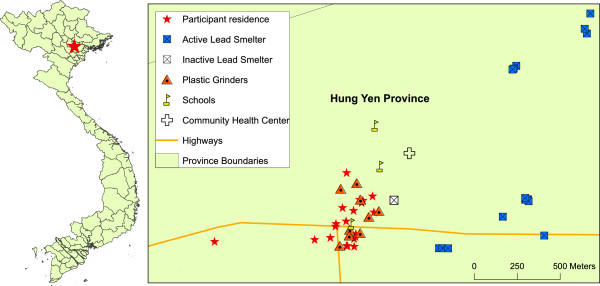
**Children in Nghia Lo, Vietnam live in close proximity to lead smelters.** Nine plastic grinders and 18 active or inactive smelters are located within three km of the Nghia Lo village.

In this study, we investigated toxic metal exposure in blood and toenail samples collected from 20 children living in Nghia Lo village, Chi Dao Commune in Northern Vietnam. Nine plastic grinders and 18 smelters were located within three km (~1.9 miles) of the village, in close proximity to schools and residential buildings (Figure 1). A single smelter was inactive at the time of this study, while the remaining 17 represent small-scale facilities were involved in active Pb smelting.

### Children in Vietnam craft villages were exposed to toxic metals

To assess children’s metal exposure in the Nghia Lo community, we measured metal levels in toenail clippings and blood Pb levels from 20 children less than five years of age. The average child’s age in this study was 2.9 years and ranged from 1.5 to 4.2 years. The sample population consisted of eleven females and nine males. The shortest recorded distance a child lived from an active smelter was 410 m. The average distance from each child’s residence to the nearest active smelter was 581 ± 188 m. Study participants’ characteristics are presented in Table 1.

**Table 1:** Characteristics of the children (n = 20) living in Nghia Lo, Vietnam

**Child age (years) mean ± SD (range)**	**2.9 ± 0.8 (1.5-4.2)**
**Average distance to nearest active Pb smelter (km), ± SD (range)**	
**Females** n (%)	11 (55%)
**Males** n (%)	9 (45%)

**Figure 2:**
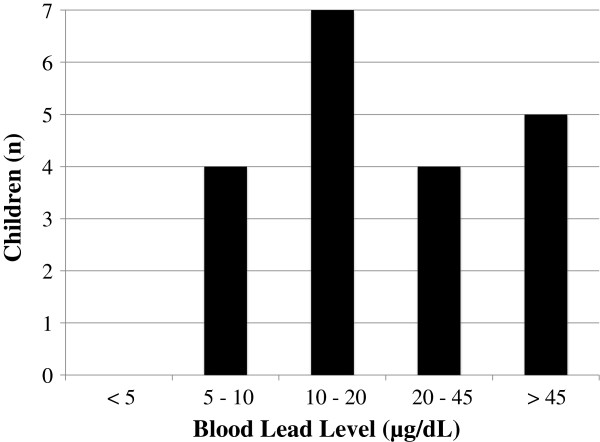
**All children had detectable blood lead levels (BLLs).** Among the 20 children in the study all had BLLs greater than 5 μg/dL, the CDC’s reference blood level for children. This included five children with BLLs greater than 45 μg/dL, the level at which chelation therapy is recommended.

Among 20 children in this pilot study, all had detectable BLLs which were above the CDC’s 5 μg/dL reference level for children [30] (Figure 2). Three of the children had BLLs above the maximum detectable level (69 μg/dL) of the portable Pb analyzer. Five of the children (25%) had BLLs above 45 μg/dL, the level above which chelation therapy is recommended [31]. Descriptive statistics of BLLs are presented in Table 2. The levels of quantifiable blood Pb ranged from 6.7 μg/dL to 62.3 μg/dL (Table 2). The geometric mean and 95^th^ percentile BLLs for individuals with detectable levels (n = 17) was 17.3 μg/dL and 62.3 μg/dL. The levels observed in the Nghia Lo children are well above the geometric mean and 95^th^ percentile reported in the Fourth Report on Human Exposure to Environmental Chemicals (NHANES IV) among children in the United States ages one to five (n = 911) which were 1.77 μg/dL and 5.10 μg/dL, respectively [32].

**Table 2:** Descriptive statistics of children’s blood and toenail metal levels (n = 20)

**Metal**^**a**^	**Mean ± stdev (μg/g)**	**Geometric mean (μg/g)**	**Minimum (μg/g)**	**95**^**th **^**percentile (μg/g)**	**Maximum (μg/g)**
Pb-B^b^	21.5 ± 16.2	17.3	6.7	62.3	62.3^a^
As-N	0.364 ± 0.312	0.289	0.109	1.09	1.46
Cd-N	0.286 ± 0.326	0.204	0.079	1.07	1.52
Cr-N	0.922 ± 0.554	0.809	0.321	2.09	2.87
Pb-N	157 ± 315	29.3	5.88	957	1120
Mn-N	7.41 ± 4.41	6.17	2.34	15.6	18.9
Hg-N	2.63 ± 7.07	1.08	0.328	17.2	32.6

^a^B denotes blood metal levels. N denotes toenail metal levels.

^b^Blood Pb units are expressed in μg/dL. Three BLLs which exceeded the 69 μg/dL maximum quantifiable level were excluded from the calculated descriptive statistics.

In addition to blood Pb, we also measured toenail levels of toxic metals including As, Cd, Cr, Pb, Mn and Hg (Table 2). Toenail levels of each metal were detected in every participant. Notably, average toenail levels of Hg, Pb, and Mn were 2.63 μg/g (maximum: 32.6 μg/g), 157 μg/g (maximum: 1120 μg/g), and 7.41 (maximum: 18.9 μg/g). These levels of Hg, Pb, and Mn were substantially higher than levels observed in previous studies. For example, in a single previous study among Saudi children the average toenail Hg level was 0.185 μg/g [33]. Two previous studies among adult populations reported average Hg toenail levels of 0.212 μg/g (geometric mean) [34], and 0.25 μg/g [35]. In previous studies of children in Kenya, Germany, and the United States, average Pb toenail levels were 26.9 μg/g [36], 8.5 μg/g [37], and 1.60 μg/g [38], respectively. In addition to levels of Hg and Pb that were higher than previously reported levels, the average toenail Mn level was considerably higher than a single previously reported level of 0.90 μg/g among children in the United States [38]. These findings highlight that markedly high levels of Pb, Hg, and Mn were detected in the toenails of the Nghia Lo children residing near craft smelting operations. Childhood exposure to Pb, Hg, or Mn is associated with diminished intellectual function [13-15,39,40].

Here we observed comparable or lower levels of As, Cd, and Cr relative to previous studies of toenail metal levels. The average toenail levels of these three metals were 0.364 μg-As/g, 0.286 μg-Cd/g, and 0.922 μg-Cr/g (Table 2). The average toenail Cr level reported here was lower than the average Cr toenail level of 2.65 μg/g among children in the United States [38]. Likewise, the average Cd level was lower than the average Cd toenail levels reported in two studies of Kenyan and German children respectively, which were 0.790 μg/g [36] and 0.457 μg/g (geometric mean) [37]. Here the average toenail As level of 0.364 μg/g was comparable to the average levels of 0.14 μg/g and 0.49 μg/g, reported in two studies of children in the United States and Australia [38,41], but substantially lower than the average level of 5.9 μg/g reported among Bangladeshi children [42]. These data show that children are exposed to detectable levels of As, Cd, and Cr; with levels similar to, or lower than, previously reported toenail levels in children.

Taken together, these findings suggest that children in battery recycling and smelting craft villages in Vietnam may significantly higher blood Pb and toenail Pb, Hg and Mn levels compared to the other measured toxic metals and compared to the levels measured in other populations. The higher metal body burden potentially results from instances of co-exposure within the craft village. This is concerning as higher body burden of Pb, Hg, and Mn in children is associated with adverse neurodevelopmental outcomes [39,40,43]. In particular, co-exposure to toxic metals has been demonstrated to have more detrimental health outcomes than single metal exposures in children [13,44] and in numerous animal studies [16-20]. Subsequent neurodevelopmental health outcomes of multiple metal exposures in local craft villages should be prioritized in future studies.

It is clear that this pilot study has limited sample size. The cross-sectional design also has inherent weaknesses, as the blood and toenail measures characterize metal levels at a single point in childhood. Blood Pb levels reflect recent exposures on the order of days, whereas toenail levels can represent longer-term exposures on the order of months. It is noted that using nail as a biomarker is a better indicator for some metals (e.g., As, Cd, Cr, and Hg) [45], whereas blood is the most appropriate biomarker for Pb. In this study, the age range of 18 months to four years was selected to characterize a developmental time period of critical susceptibility. The elevated metal measures in nail biomarkers are particularly alarming indicators of early childhood metal exposure and additional studies should collect multiple samples throughout childhood.

### Toxic metal levels in blood and toenails were correlated

To better understand co-exposure to toxic metals, we examined the pairwise relationships between metal levels measured in children’s blood and toenail samples (Table 3). Spearman’s rank correlation showed a statistically significant positive correlation (p < 0.05; r = 0.65) between Pb levels measured in the blood (μg/dL) and Pb levels in toenail samples (μg/g) (Table 3). No other relationships between blood Pb and toenail metal levels were statistically significant. These findings potentially indicate both chronic and acute Pb exposure as reflected by nail and blood levels, respectively [46]. Children may be exposed to Pb through a variety of smelting and non-smelting related activities such as direct contact with smelting materials; contact with Pb residues remaining on parents’ work clothing; or via contaminated air, water, food, or soil. The evidence of high Pb exposure warrants further investigation as to the primary mode of exposure in Vietnam battery recycling craft villages in the effort to reduce childhood exposures.

**Table 3:** Spearman’s correlation (r) for toxic metal levels in children’s toenails (n = 20) and blood (n = 17)

**Metal**^**a**^	**Cd-N**	**Cr-N**	**Pb-N**	**Mn-N**	**Hg-N**	**Pb-B**
As-N	0.59*	0.07	0.42	0.58*	−0.10	0.00
Cd-N		0.26	0.66*	0.60*	0.03	0.04
Cr-N			0.02	0.72**	0.26	−0.43
Pb-N				0.33	0.02	0.65*
Mn-N					0.01	−0.30
Hg-N						−0.32

*p < 0.05.

**p < 0.001.

^a^B denotes blood metal levels. N denotes toenail metal levels.

Among toenail levels, our analysis revealed statistically significant (p < 0.05; r > 0.45) positive Spearman’s correlations between Mn and Cr, Pb and Cd, Mn and Cd, As and Cd, and As and Mn. The two strongest relationships were observed between Mn and Cr (r = 0.72, p < 0.001) as well as Pb and Cd (r = 0.66, p < 0.05). Hg levels had weak associations with the other metal levels. In addition to smelting practices, several other sources of metals may contribute to co-exposure in the Nghia Lo community. For example, elevated levels of As and Mn have been found in drinking water sources in the Red River Delta [24,25,27]. A previous study found that 27% and 44% of groundwater wells exceeded the World Health Organization (WHO) guideline values for As (10 μg/L) and Mn (0.4 mg/L), respectively [24,47,48]. Drinking water As levels in Vietnam range up to 3050 μg/L [25,27], a level greater than 300 times the recommended WHO drinking water standard [47]. The biomonitored levels of As were lower in this group of children when compared to previous studies with high level As contamination; however, low-level and multi-metal exposure in childhood remains an important consideration for future health outcomes. The traditional Vietnamese diet also includes a high proportion of local seafood, which has been documented to contain methylmercury levels up to 0.5 μg/g [49,50]. Compared with the United States EPA guideline of consumption of 0.1 μg/kg of methylmercury [51], dietary Hg represents a potential important alternate source of exposure. Other craft operations, including leather tanning, are documented sources of Hg and Cr exposure in craft villages [52-55]. For example, Cr salts used in tanning practices in Vietnam produce large amounts of solid and liquid toxic metal waste [55,56]. Although levels of As and Cr were relatively low in the toenail samples, multiple toxic metal biomarkers may be of interest in future studies to understand metal mixtures. The present study did not collect information on individual’s diet, other craft operations in Nghia Lo, or measure toxic metals other than Pb in blood; these are relevant potential sources or biomarkers of metal exposure that should be considered in future studies.

### Distance to nearest active smelter and metal levels were associated

Lastly, we examined the relationship between blood Pb and toenail metal levels to children’s age, sex, and distance to the nearest active smelter using linear regression (Table 4). On average, males had a higher Pb level in both blood and toenails (BLL: 22.35 μg/dL, Pb-N: 86.8 ng/g) than females (BLL: 20.8 μg/dL, Pb-N: 54.3 ng/g), but no statistically significant association was found between Pb level and sex. Similarly, no significant relationship with child’s age was observed between blood Pb or any toenail metal levels. Our findings do not demonstrate a statistically significant association with child’s age or sex. The lack of association with age may have been a result of unmeasured confounding due to the small sample size, or the relatively limited age range (1.5 to 4 years). Additional factors that affect metal body burden include child’s body mass index, socioeconomic status, duration of residence, parental smoking status, and occupation. These covariates will aid in better characterizing the demographics of smelting craft village populations and should be considered in future studies.

**Table 4:** Beta estimates (95% confidence intervals) calculated from unadjusted linear regression analyses between toxic metal levels and distance to nearest active smelter, child’s age, or sex

**Metal**^**a**^	**Distance to nearest active smelter**		**Age**	**Sex**^**c**^
	**β (95% CI)**	**Fold-change (% change)**^**b**^	**β (95% CI)**	**β (95% CI)**
Pb-B	−1.11 (−2.21, -0.01)^*^	2.16 (116%)	−0.17 (−0.59, 0.24)	−0.14 (−0.74, 0.47)
As-N	−0.29 (−1.47, 0.89)	1.22 (22%)	0.10 (−0.30, 0.51)	−0.32 (−0.90, 0.26)
Cd-N	−0.35 (−1.34, 0.64)	1.28 (28%)	−0.08 (−0.43, 0.26)	−0.39 (−0.86, 0.08)
Cr-N	0.97 (0.11, 1.84)^*^	0.51 (−49%)	−0.05 (−0.38, 0.29)	0.22 (−0.26, 0.70)
Pb-N	−1.34 (−3.65, 0.96)	2.54 (154%)	−0.07 (−0.90, 0.75)	−0.43 (−1.61, 0.75)
Mn-N	0.39 (−0.75, 1.53)	0.76 (−24%)	−0.07 (−0.47, 0.33)	−0.11 (−0.69, 0.46)
Hg-N	2.39 (0.80-3.98)^*^	0.19 (−81%)	0.04 (−0.63, 0.71)	0.69 (−0.23, 1.61)

^*^p < 0.05.

^a^B denotes blood metal levels. N denotes toenail metal levels.

^b^Metal levels and the distance to the nearest active smelter were natural-log transformed. The fold-change values represent the exponentiated beta value (0.5^β^) and corresponding percent change in BLL when the distance to smelter is decreased by half.

^c^Females served as the referent group.

The relationship between BLLs and the distance between a child’s place of residence to the nearest active smelter showed a statistically significant (p < 0.05) negative correlation. Specifically, a 50% reduction in the distance to the nearest active smelter was associated with a 2.16 fold-change or 116% increase in BLL. In fact, it is possible that due to our selection criteria these results may underestimate the relationship between distance and BLL. In addition, it is possible that children living farther from a smelter may have had parents who worked in Pb smelting, whereas children living closer to a smelter may have had parents who did not.

Toenail levels of As, Cd, and Pb were also negatively correlated with the distance to the nearest smelter, but were not found to be statistically significant. Especially relevant to the toenail biomarker, which is indicative of long-term exposure, it is important to note that there may have been historic exposure from the inactive smelter that was located closest to the village. The association between toenail levels of Cr and Hg and distance to the nearest active smelter showed statistically significant positive relationships for Cr and Hg (p < 0.05), meaning that children living farther away from Pb smelting operations had higher toenail levels of Cr and Hg. However, exclusion of the child with the furthest distance to smelter resulted in a negative, and non-statistically significant relationship between distance to nearest smelter and nail levels of Cr and Hg (data not shown). These results may also be influenced by unmeasured confounding, and should be substantiated with a larger study sample.

## Conclusions

Battery smelting craft practices in rural Vietnamese communities continue to increase in number due to the lucrative economic benefits. Data from this cross-sectional pilot study indicate that children are not adequately protected from resultant toxic metal exposures. Overall, the findings show that children living in a battery recycling community had elevated levels of blood Pb and elevated levels of Pb, Hg, and Mn in toenail samples. Increased levels of Pb, Hg and Mn are known to cause adverse neurodevelopmental outcomes in humans, and children are especially susceptible. In addition, we find that children are co-exposed to multiple other toxic metals including As, Cd, and Cr, within the craft village setting. Metal exposure may arise directly as a result of smelting activities or other potential sources. This research emphasizes the need for both increased biomonitoring of metal exposure and public health strategies in these communities to alleviate children’s exposure. Future studies to assess the adverse neurodevelopmental affects of multiple metal exposures among children living in smelting craft villages are needed.

## Abbreviations

As: Arsenic; BLL: Blood lead level; Cd: Cadmium; Cr: Chromium; CDC: Centers for disease control and prevention; GPS: Global positioning system; Hg: Mercury; IQ: Intelligence quotient; LOD: Limit of detection; Mn: Manganese; NHANES IV: Fourth report on human exposure to environmental chemicals; Pb: Lead; WHO: World Health Organization.

## Competing interests

The authors declare that they have no competing interests.

## Authors’ contributions

RCF and JBK conceived of and designed the study. VN collected the data. APS and SKM carried out the analyses. APS, SKM, and RCF wrote the manuscript. All authors read and approved the final manuscript.

## Pre-publication history

The pre-publication history for this paper can be accessed here:

http://www.biomedcentral.com/1471-2458/14/114/prepub
